# What will it take for behavior to change? Findings from a baseline evaluation of an intervention to reduce antimicrobial use among smallholder poultry farmers in Zimbabwe

**DOI:** 10.3389/fvets.2026.1924591

**Published:** 2026-09-01

**Authors:** Patience Chenayimoyo Mushanawani, Webster Mavhu, Chenai Sipiwe Majuru, Canaan Tinashe Hodobo, Tsamwi Naume Gurira, Emily Waniwa, Kudzai Nyoka, Grace Maparo, Wasarirevu Mawere, Johannes Mubvuta, Iredale Mutengwa, Philip Zvorufura, Tariro Madzivanyika, Mackwellings Phiri, Tafadzwa Charumbira, Erica Westwood, Sarah von Humboldt-Scotland, Cèlia Ventura-Gabarró, Ahmad Wesal Zaman

**Affiliations:** 1 Department of Veterinary Technical Services, Ministry of Agriculture, Mechanisation and Water Resources Development, Harare, Zimbabwe; 2 Centre for Sexual Health and HIV/AIDS Research (CeSHHAR), Harare, Zimbabwe; 3 Department of International Public Health, Liverpool School of Tropical Medicine, Liverpool, United Kingdom; 4 Independent Consultant, Harare, Zimbabwe; 5 Malawi Liverpool Wellcome Clinical Research Programme, Blantyre, Malawi; 6 Poultry Farmers Association of Zimbabwe, Harare, Zimbabwe; 7 International Centre for Antimicrobial Resistance Solutions (ICARS), Copenhagen, Denmark

**Keywords:** antimicrobial resistance, antimicrobial use, behavior change, COM-B, one health, poultry farmers, women, Zimbabwe

## Abstract

**Introduction:**

Antimicrobial resistance (AMR) occurs when pathogens become immune to drugs designed to eliminate or suppress their growth. AMR is accelerated by the inappropriate use of antimicrobials, a common practice among small-medium scale poultry farmers. We conducted a mixed-methods baseline exploration for an intervention to strengthen biosecurity practices and prudent antimicrobial use (AMU) among small-scale poultry farmers in Zimbabwe, to identify key baseline behaviors and their determinants as well as potential intervention entry points.

**Methods:**

December 2024 to April 2025, we iteratively conducted 45 qualitative interviews and six conversation events with purposively selected farmers. To explore the underlying drivers of behavioral practices, the Capability, Opportunity, Motivation, and Behavior (COM-B) model for behavior change was applied as an organizing framework.

**Results:**

Overall, five key behaviors relevant to biosecurity and AMU in the poultry sector emerged from the analysis: (1) incomplete adoption of biosecurity practices; (2) self-diagnosis and self-treatment of poultry disease; (3) prophylactic antibiotic use; (4) non-observance of withdrawal periods; and (5) unsafe disposal of dead birds and poultry waste. In addition, a cross-cutting determinant, gendered distribution of household and poultry labor, was found to influence multiple behaviors by shaping female farmers' access to time, resources and opportunities to implement recommended practices. Analysis of these behaviors through the COM-B framework revealed how capability, opportunity and motivation interacted to shape farmers' practices.

**Conclusion:**

AMU among poultry farmers is closely linked to a range of behaviors undertaken within production systems characterized by various structural and social factors. Interventions need to address the underlying drivers of AMU rather than its symptoms alone. Intervention components could include practical biosecurity training, low-cost biosecurity innovations, training on withdrawal periods, community-led disposal systems and, household workload-sharing strategies. The resultant intervention will need to be evaluated for effectiveness.

## Introduction

Antimicrobial resistance (AMR) occurs when bacteria, fungi, parasites and viruses evolve over time to survive exposure to drugs designed to eliminate or suppress their growth (1, 2). This otherwise evolutionary process has been significantly accelerated by human actions including the misuse and overuse of antimicrobials in humans, animals and plants (1, 3). AMR is now recognized as one of the most urgent global health threats (2). A global estimate of the burden of bacterial AMR in 2019 found that of the roughly 8.9 million deaths due to bacterial infections that year, 1.27 million deaths were attributable to, and 4.95 million deaths were associated with, AMR (4).

Ninety percent of AMR deaths were estimated to have occurred in low-and middle-income countries (LMICs) (4), and the burden of AMR in Africa is one of the highest in the world (5–7). These settings have high prevalence of infectious diseases; limited access to clean water, sanitation and hygiene; insufficient quality healthcare, including limited infection prevention and control; limited access to diagnostics, and frequent and unrestricted antimicrobial use (8–10), factors which drive AMR. Indeed, both the use of antimicrobials and transmission of resistant infections are heavily influenced by the social and structural conditions that are shaped by complex social structures, including gender and equity dynamics, which influence unequal labor burdens (10–12). Research has also shown that antimicrobial use (AMU) ensures continued productivity in humans and livestock, and thus serves as a ‘quick fix' for illnesses that stem from entrenched intersectional issues, including poor sanitation, inequality and weak health systems (13, 14).

Without urgent action, AMR could cost the global economy up to USD 100 trillion by 2050 and the number of AMR-related deaths could rise to 10 million annually (5). As the crisis deepens, calls for coordinated, cross-sectoral actions have led to the rise of the One Health approach, an integrated, unifying strategy that seeks to sustainably balance and optimize the health of people, animals and ecosystems (10, 15). It acknowledges that the health of humans, domestic and wild animals, plants and the environment are closely linked and interdependent, particularly in relation to AMR (16). However, sector-specific and cross-sectoral solutions needed to address AMR too often lack the multidisciplinary approach needed for a holistic response to this challenge (10, 15).

Zimbabwe has witnessed a substantial increase in its poultry populations, within a context of various structural, socio-economic and behavioral factors that drive widespread inappropriate antibiotic use. This practice creates an environment that is highly conducive to the emergence and proliferation of antimicrobial resistant pathogens (8, 10). It is against this background that a multi-stakeholder initiative—‘Strengthening biosecurity practices in the poultry value-chain in Zimbabwe, to reduce the use of antibiotics'—was launched in Zimbabwe in 2024. The project's overall goal is to reduce AMU through improving farm-level biosecurity (e.g., controlling farm visitors, protecting feed from pests).

As a first step, we conducted a mixed-methods baseline evaluation to assess current biosecurity practices and their determinants. This approach is particularly relevant in smallholder farming systems where access to veterinary services, quality livestock inputs, financial resources and labor may strongly influence disease-management decisions and AMU practices. Although inappropriate AMU is often attributed to knowledge deficits among farmers, relatively little is known about how capability, opportunity and motivation interact to shape poultry-health behaviors in smallholder production systems in Africa. Here, we report on these aspects.

## Materials and methods

### Study design

We adopted a mixed-methods approach combining qualitative individual interviews with participatory community conversation events as part of responsive dialogue in three Zimbabwean districts: Goromonzi, Seke and Zvimba, and two villages in each district aiming to ensure variation between both districts and villages (e.g., different agro-ecological zones; market access levels). The baseline evaluation was conducted in all six villages.

### Data collection, processing and analysis

#### Individual qualitative interviews

From December 2024 to April 2025, 45 smallholder poultry farmers were selected to take part in individual qualitative interviews. To comprehensively understand the drivers of farmer behaviors, the interview guide probed a wide range of topics including: routine poultry management (e.g., housing and feeding), recent disease episodes and treatment responses, biosecurity awareness, antibiotic usage patterns, resource access, decision-making influences within the household and, perceived obstacles to raising healthy chickens.

Sampling was purposive with a maximum of 15 farmers per district, as well as equitable representation of men and women. Care was taken to include participants from various social backgrounds, ensuring the inclusion of disadvantaged, vulnerable and marginalized women and men (e.g., widowed, disabled, less educated). Timing of interviews also ensured the interview would not clash with household duties.

All discussions were led by trained Zimbabwean researchers in private spaces and lasted up to an hour. Most discussions were in Shona, the participants' language and a few were in English. With the participants' permission, the discussions were audio-recorded; hand-written notes were taken as back up. The recorded interviews were transcribed and translated verbatim into English (where necessary). To ensure validity, all transcripts were read against their respective audio files to confirm their accuracy.

We described the sociodemographic characteristics of participants who took part in the individual interviews by district. The interview coding process began with two researchers (PCM and GM) independently coding the same transcripts using a coding framework based on the topic guide and adding codes as analysis progressed. Afterwards, they met with a more senior researcher (WeM) to compare their coded transcripts; discrepancies were resolved through discussion. A third (*n* = 15) of transcripts was coded by both coders, with 12 showing consistency between the two researchers. The 45 transcripts were then entered into NVivo 14 (QSR International, Melbourne, Australia) and fully coded using the modified coding framework. As inter-coder reliability had been achieved with the initial 15 transcripts, the subsequent coding was shared between the two researchers. The team still met regularly to develop a common understanding of the codes and their application and to discuss emerging patterns. Codes were grouped and emerging themes were identified and supported with verbatim quotes. Quotes from the individual interviews were linked to each participant's sociodemographic data collected at the start of the interview to enable a more nuanced appreciation of issues.

#### Conversation events as part of responsive dialogue

From March to April 2025, a total of six conversation events were held (n=2 per district, n=1 per village). These were a series of interconnected dialogues, each with a different focus, from introducing AMR and unpacking participants' lived experiences of AMR challenges, to participants jointly developing ideas to address these challenges (called co-ideation), and moving to participants co-creating context-specific and feasible solutions that can bring about sustainable change at a local and policy level. In these half-day interactive group dialogues (~*n* = 25 participants per group), participants listed, ranked and voted to identify the top five challenges. For each top challenge, the group discussed and listed any solutions they had tried or could propose. The contributions were recorded through notes and flipcharts and were later transcribed under the headings: “Challenges” and “Proposed solutions”.

**Figure 1:**
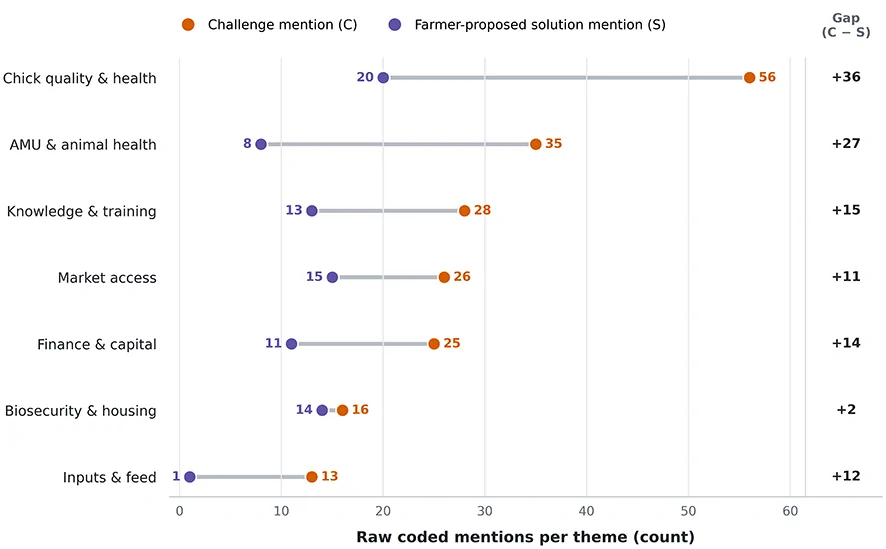
This plot shows the challenges-solutions gap for each theme. The x-axis presents raw counts of theme-coded mentions; no normalization was applied. When the orange dot lies far to the right of the purple dot, this indicates frequently-raised challenges for which farmers had few solutions.

For analysis, the R script identified text appearing under the “Challenges” and “Proposed solutions” headings and divided it into discrete statements using paragraph breaks, bullet points, semicolons, hyphens and similar delimiters. The statements were standardized by removing excess spacing and then classified, using case-insensitive keyword dictionaries, into seven themes informed by a baseline analysis: Finance and Capital, Market Access, Chick Quality and Health, AMU and Animal Health, Biosecurity and Housing, Knowledge and Training, and Inputs and Feed. A statement could be assigned to more than one theme if it contained keywords from multiple thematic dictionaries; unmatched statements were classified as “Other” and excluded from the gap figure (Figure 1). Theme-level challenge and solution mentions were then counted across all six sites. The problem–solution gap for each theme was calculated as the difference between the raw number of challenges mentions and that of solutions mentions. These values therefore represent coded statement–theme mentions rather than numbers or proportions of individual participants. A large problem–solution gap suggests a problem that farmers recognize but cannot solve on their own.

#### Capability, opportunity, motivation, and behavior (COM-B) analytical approach

Behavioral frameworks such as the Capability, Opportunity, Motivation, and Behavior (COM-B) model (17) enable researchers to move beyond explanations based solely on knowledge deficits and examine how structural, economic and social conditions shape behavior. To explore the underlying drivers of behavioral practices, the COM-B model for behavior change (17) was applied as an organizing framework. COM-B specifies three conditions: capability (C), opportunity (O) and, motivation (M) that are essential for changing behavior (B). Capability implies one's psychological and physical ability to participate in an activity; Opportunity refers to external factors that make a behavior possible and; Motivation implies the conscious/unconscious cognitive processes that direct and inspire change (17). Themes identified from individual interviews were systematically interpreted according to how farmers' Capabilities, Opportunities and Motivations influenced each specific behavior (see Table 1).

**Table 1:** COM-B analysis of key poultry biosecurity and antimicrobial use behaviors among smallholder poultry farmers in Zimbabwe.

COM-B domain	1. Incomplete adoption of biosecurity practices	2. Self-diagnosis and self-treatment of poultry disease	3. Prophylactic antibiotic use	4. Non-observance of withdrawal periods	5. Unsafe disposal of dead birds and waste	Cross cutting: gendered distribution of household and poultry labor
Psychological capability	Partial understanding of disease transmission pathways and biosecurity hygiene practices	Limited ability to recognize disease syndromes and distinguish between conditions requiring different treatments	Learning from previous experiences that antibiotics appear to reduce losses	Limited awareness of withdrawal periods and drug-specific requirements	Limited understanding of pathogen transmission and biosecurity risks associated with disposal practices	Women demonstrate comparable poultry-management knowledge to men. Women's biosecurity-related barriers are rooted in structural and social constraints (time poverty, competing domestic responsibilities) that prevent them from acting on existing knowledge.
Physical capability	Limited ability to consistently perform recommended biosecurity practices due to competing demands and labor constraints	Limited practical diagnostic skills and inability to confirm diagnoses	Limited capacity to implement alternative disease-prevention approaches when resources are constrained	Limited ability to keep birds longer when treatment has occurred and resources are scarce	Limited technical skills and equipment for safe disposal practices	Physical fatigue and workload burden reduce ability to consistently implement biosecurity measures amongst women
Physical opportunity	Limited access to disinfectants, footbaths, protective equipment, improved housing and capital	Limited veterinary access, high service costs and poor availability of professional support	Poor chick quality, limited access to vaccinated chicks and inadequate preventive health services	Feed costs, cash-flow pressures and economic constraints	Lack of accessible disposal infrastructure or practical alternatives	Women's time poverty due to domestic responsibilities, childcare and poultry management
Social opportunity	Hospitality norms, visitor expectations and cultural obligations make visitor restrictions difficult	Reliance on chick suppliers, agrovet shops, neighbors and peer networks for disease-management advice	Advice from suppliers, peers and informal networks may normalize preventive antibiotic use	Market pressures and expectations regarding income generation influence decisions to sell birds early	Religious beliefs, household norms and practical traditions shape disposal practices	Gendered expectations around household chores, childcare and poultry care place disproportionate responsibilities on women
Reflective motivation	Farmers recognized the importance of preventing disease and demonstrated willingness to adopt feasible biosecurity measures to protect flock health and livelihoods	Confidence in self-diagnosis and belief that experiential knowledge can effectively guide treatment decisions	Deliberate use of antibiotics to protect flock investments and minimize economic losses	Conscious decision to prioritize immediate income needs over adherence to withdrawal periods	Disposal practices viewed as practical and economically sensible within local contexts	Men and women recognize the unequal burden on women and acknowledge the need for change
Automatic motivation	Repeated disease outbreaks and previous failures reduce confidence in the effectiveness of biosecurity measures	Habitual reliance on familiar treatment practices and trusted advice networks	Fear of chick mortality, anxiety about losses and routine use of antibiotics as “insurance”	Immediate financial pressures create urgency to sell birds regardless of withdrawal periods	Habitual disposal practices reinforced through long-standing cultural and household routines	Established social norms and ingrained gender roles reinforce existing labor divisions

#### Positionality statement

All authors have been involved in initiatives to strengthen biosecurity practices and prudent AMU in Zimbabwe and other settings. This may have influenced their research “tone”. Additionally, some are affiliated with a government department, and may have been perceived by participants as “enforcers” as opposed to “researchers”. This may have resulted in participants attempting to present some behaviors e.g., inappropriate antibiotic use and withdrawal-period violations as structural issues rather than personal decisions, a recognized tendency in behavior-related research (18, 19).

## Results

### Participants' socio-demographic characteristics

Forty-five participants took part in individual interviews. Of these, 32 (71.1%) were female and 13 (28.9%) were male, reflecting the general gender composition of small-medium scale farmers in Zimbabwe (20). Median age was 45 years (range 22–68 years). About a quarter (11/45, 24.4%) had primary or some secondary schooling, about half (23/45 (51.1%) had completed ordinary level schooling and 2 (4.4%) had advanced level schooling. A fifth (9/45, 20%) had some tertiary education. Nearly all participants (44/45, 97.8%) were small-scale farmers, keeping 25–200 chickens (small-scale farmers are defined as those keeping 25–999 chickens), including a female farmer (46yrs, #19) with 700 chickens. The only medium-scale farmer (male, 46yrs #20) had 5,000 chickens. Conversation events participants were also largely female (~70%).

### Key behavioral domains

Overall, five key behaviors relevant to biosecurity and AMU in the poultry sector emerged from the analysis: (1) incomplete adoption of biosecurity practices; (2) self-diagnosis and self-treatment of poultry disease; (3) prophylactic antibiotic use; (4) non-observance of withdrawal periods; and (5) unsafe disposal of dead birds and poultry waste. In addition, a cross-cutting determinant, gendered distribution of household and poultry labor, was found to influence multiple behaviors by shaping farmers' access to time, resources and opportunities to implement recommended practices. Analysis of these behaviors through the COM-B framework revealed how capability, opportunity and motivation interacted to shape farmers' practices. We discuss each behavior and its underlying determinants below.

## Incomplete adoption of biosecurity practices

The adoption of recommended biosecurity practices was frequently constrained by interacting capability, opportunity and motivation factors. Although most farmers generally recognized the importance of disease prevention and expressed willingness to implement protective measures, biosecurity practices were often implemented inconsistently or only partially. From a capability perspective, some farmers demonstrated limited understanding of disease transmission pathways and the mechanisms through which biosecurity measures prevent infection. While participants were often aware of recommended practices such as hand disinfection and footbaths, knowledge regarding the rationale underpinning these measures appeared inconsistent. In conversation events, “lack of knowledge” was the third most-mentioned problem (*n* = 28 challenge; *n* = 13 solution mentions—a gap of 15), highlighting how farmers recognized this challenge but could not simply self-solve it (Figure 1).

Physical opportunity emerged as a major constraint on biosecurity implementation. Many farmers reported lacking the financial resources required to purchase disinfectants, footbaths and other biosecurity inputs. As one participant explained: “*I am aware that one should use a disinfectant, either on their hands or in the footbath but I use ash [wood] because I cannot afford these chemicals”* (Female, 40 yrs #38). As illustrated later, limited financial resources influenced several other practices. In addition, structural challenges such as widespread chicken theft influenced housing practices. To protect their birds, some farmers brought chickens into their homes at night, thereby limiting their ability to maintain recommended biosecurity procedures. As one farmer noted: “*You know if you are keeping them [chickens] in the house, you will not be able to practice certain measures like using the footbath”* (Female, 52 yrs #04).

Social opportunity also influenced biosecurity behaviors. Participants described situations where visitors were allowed into poultry areas without appropriate precautions because restricting access was perceived as impolite or inconsistent with local norms of hospitality. These findings suggest that biosecurity practices were shaped not only by material resources but also by social expectations and cultural obligations.

Furthermore, gendered labor arrangements influenced the implementation of biosecurity practices, with women often balancing poultry management alongside multiple household responsibilities. A participant narrated, “*I wake up in the morning and sweep the house. I then prepare stuff for school kids, preparing their “breakfast” and so on. As soon as I finish these tasks, I then enter the fowl runs. When I come out of these, I start cleaning the yard and then do other tasks. But, by then, I would have cleaned the fowl runs, fed chickens and then every 3 to 4 hours, I will check if the chickens still have some drinking water”* (Female, 41 yrs #01). The disproportionate labor burden was acknowledged by male participants. ‘*When it comes to chores, we see that one “gender” has many roles and the other “gender” has only a few'* (Male, 58 yrs #16). Importantly, these many chores compromised biosecurity practices. As one female farmer put it, ‘*…I won't even wash my hands. I will just enter the fowl run and add some water'* (Female, 51 yrs #15). From a reflective motivation perspective, both men and women recognized the unequal burden on women and acknowledged the need for change.

Taken together, the findings suggest that incomplete adoption of biosecurity practices was driven less by a lack of motivation and more by constraints in physical and social opportunity, compounded by selected capability gaps. Farmers frequently demonstrated willingness to engage in preventive practices, but their ability to do so was limited by resource constraints, competing demands and contextual realities beyond their direct control.

## Self-diagnosis and self-treatment of poultry diseases

Self-diagnosis and self-treatment emerged as a common disease-management behavior among most poultry farmers. Rather than seeking professional veterinary advice, many farmers relied on their own observations, previous experiences and informal advice networks to diagnose and treat disease outbreaks. From a psychological capability perspective, farmers frequently demonstrated limited ability to accurately identify disease syndromes and distinguish between conditions requiring different treatment approaches. Disease recognition was often based on visible symptoms, previous experiences or informal diagnostic practices, including post-mortem examinations. For example, one farmer explained: “*Like the last batch, we treated the chickens when they began to show some signs of coccidiosis. They were excreting ‘red' droppings… You actually ‘know' that when a chicken develops coccidiosis, you use ESB3 or Teranox”* (Female, 46 yrs #03). Another participant described diagnosing Newcastle disease after observing physical changes during an informal post-mortem examination: “*When I ripped the dead chicken open, I noticed that it was watery inside and the liver was swollen and I ‘knew' it was Newcastle”* (Female, 22 yrs #17). These practices suggest that while farmers possessed practical experiential knowledge, diagnostic capability was often limited and may have contributed to misdiagnosis and inappropriate treatment decisions.

Physical opportunity constraints were also prominent. Many participants reported limited access to affordable veterinary services and professional animal-health support. In the absence of accessible veterinary care, farmers frequently relied on chick suppliers, agrovet shops and drug sellers for disease-management advice. As one farmer explained: “*We call the chick supplier and say the chickens are experiencing the following symptoms and they say, ‘You need to use Bremamade'. They actually tell us which medications to use”* (Male, 55 yrs #05). This suggests that treatment decisions were often shaped by the availability and accessibility of informal service providers rather than formal veterinary systems.

These findings were further supported by the conversation events, where AMU and animal health emerged as one of the most frequently-cited challenges facing poultry farmers. Despite widespread concerns regarding disease outbreaks and treatment failures, participants proposed relatively few viable solutions, resulting in one of the largest challenge–solution gaps identified during the discussions (Figure 1). This suggests that farmers recognized the limitations of current disease-management practices but lacked access to effective alternatives and professional support.

Social opportunity further influenced disease-management practices. Participants described relying heavily on peer networks, family members, chick suppliers and community knowledge when making treatment decisions. Traditional remedies formed an important component of these informal knowledge systems and were often viewed as legitimate alternatives to biomedical approaches. One farmer explained: “*After 3 days of obtaining your chicks, you give them* ***mutiti*** *(lucky-bean tree). Whenever they develop a flu, you give them* ***gavakava*** *(aloe vera) and* ***mhiripiri*** *(chilies). We witnessed our grandmothers and mothers using these”* (Female, 50 yrs #08). These findings illustrate how disease-management practices were embedded within broader social and cultural systems of knowledge transmission.

Automatic motivation appeared to operate through habitual reliance on familiar treatment approaches and trusted sources of advice. Repeated use of particular medicines, remedies and advice networks appeared to create routine patterns of disease management that were reproduced during subsequent disease outbreaks.

The findings suggest that self-diagnosis and self-treatment were shaped by interacting capability, opportunity and motivation factors.

## Prophylactic antibiotic use

The prophylactic administration of antibiotics to healthy chicks or flocks emerged as a common disease-prevention practice among most farmers and across all farmer groups. Farmers reported frequently administering antibiotics before the onset of disease as a strategy to reduce mortality and protect flock productivity. Rather than being viewed solely as treatments for illness, antibiotics were often perceived as preventive tools that could safeguard birds against anticipated health risks.

From a psychological capability perspective, farmers drew heavily on experiential learning when making decisions about antibiotic use. Many participants reported that repeated observations of reduced mortality following antibiotic administration reinforced the perception that prophylactic use was beneficial. As one farmer explained: “*There is this medication, ‘Cloves'. I found it very helpful. They say it treats all diseases…I realized that if I administered it frequently, I would not experience chicken deaths and some ‘losses'. Actually, it boosts my business”* (Female, 22 yrs #17). Such accounts suggest that farmers' decisions were informed not only by formal knowledge but also by practical experience accumulated through poultry production.

Physical opportunity constraints also played an important role in shaping prophylactic antibiotic use. Farmers repeatedly linked disease prevention practices to broader structural challenges, particularly poor chick quality and limited access to vaccinated chicks. During both interviews and conversation events, participants described receiving weak or unhealthy chicks and experiencing high mortality shortly after purchase. In this context, prophylactic antibiotic use functioned as a compensatory strategy to address perceived deficiencies within the poultry supply chain. The substantial challenge–solution gap associated with chick quality observed in conversation events (Figure 1) suggests that farmers perceived this problem as largely beyond their control, reinforcing reliance on antibiotics as a compensatory disease-prevention strategy.

Social opportunity influenced prophylactic antibiotic use through interactions with chick suppliers, agrovet shops and informal advice networks. Participants reported receiving recommendations to administer antibiotics before disease occurred, often from suppliers who framed prophylactic use as necessary for successful poultry production. One participant noted: “*I used to use antibiotics after my chickens had fallen ill but I was advised that it was often ‘too late' and I needed to give them beforehand. Since then, I have not experienced any problems”* (Male, 46 yrs #20). These findings suggest that social networks and trusted information sources played an important role in shaping perceptions of appropriate disease-prevention practices.

Taken together, the findings suggest that prophylactic antibiotic use was not driven by a single COM-B domain but by the interaction of capability, opportunity and motivation factors. Farmers' experiences, constrained access to preventive alternatives, influence from trusted advice networks, and concerns about flock survival, collectively contributed to the normalization of prophylactic antibiotic use.

## Non-observance of withdrawal periods

The non-observance of antibiotic withdrawal periods emerged as a common self-reported practice among most poultry farmers. Participants reported selling eggs or chickens before antibiotics had fully cleared from the animals' systems, a practice that carries the risk of antimicrobial residues entering the food chain. While some instances reflected knowledge gaps, many farmers described making deliberate decisions to prioritize economic survival over adherence to recommended withdrawal periods. From a psychological capability perspective, awareness and understanding of withdrawal periods varied among participants. Some farmers demonstrated limited knowledge regarding the purpose of withdrawal periods, the risks associated with antimicrobial residues and the required waiting periods for specific drugs. For example, one participant explained*: “We are not aware of the dangers of selling chickens within 2 weeks of giving them antibiotics; we need to be taught”* (Female, 56 yrs #31). These findings suggest that capability deficits contributed to non-compliance among some farmers.

However, physical opportunity constraints emerged as a particularly important driver of this behavior. Farmers frequently described operating under severe financial pressures, with feed costs, treatment expenses and household needs creating immediate demands for income. Under these conditions, delaying the sale of birds until withdrawal periods had elapsed was often perceived as economically unfeasible. As one participant explained: “*We know that it is important [to observe withdrawal period]. However, I will also be looking at my ‘budget'. There won't be money anymore to buy feed”* (Female, 52 yrs #04). These accounts suggest that compliance with withdrawal recommendations was often constrained by limited financial resources rather than an absence of knowledge alone. Conversation events findings provided additional evidence for the role of economic constraints in shaping poultry-health behaviors. Farmers consistently identified limited access to capital and challenges in marketing birds and eggs as major concerns. These broader financial pressures help explain why compliance with withdrawal periods was often difficult in practice, even among farmers who understood the associated risks.

Reflective motivation was evident in the way farmers consciously weighed competing priorities. Many participants recognized the importance of withdrawal periods and the potential risks associated with antimicrobial residues but simultaneously considered the economic consequences of delaying sales. One participant noted: “*We all know about the ‘withdrawal periods', we don't want to lie. But we also know that a 5-week-old chicken is a duck [implying quite big]. When chickens fall ill at 5 weeks, you treat and sell them at the same time”* (Female, 46 yrs #19). This suggests that non-observance was often a deliberate trade-off between food-safety considerations and economic necessity. Automatic motivation further reinforced this behavior through the immediacy of financial pressures and the routine need to maintain cash flow. The urgency of covering feed costs, replacing stock and meeting household expenses created powerful incentives to sell birds as soon as market opportunities arose.

In a nutshell, the findings suggest that non-observance of withdrawal periods was shaped by interacting capability, opportunity and motivation factors. While knowledge deficits contributed to the behavior among some farmers, economic constraints emerged as the dominant influence.

## Unsafe disposal of dead birds and waste

Unsafe disposal of dead birds and poultry waste emerged as a common practice among most farmers. Participants reported disposing of dead birds through a range of methods, including feeding them to people or animals, practices that may increase the risk of pathogen transmission. From a psychological capability perspective, many participants demonstrated limited understanding of the pathways through which diseases can spread from infected birds to animals, humans or the wider environment. Disposal decisions were often based on visible signs of illness rather than an understanding of microbial transmission risks. For example, one participant explained: “*Our apostolic faith does not allow us to eat animals that die on their own and so we give the dead chickens to some boys who offer to perform menial jobs like cutting firewood in exchange for these”* (Female, 50yrs #08). Another participant reported feeding dead chickens to dogs after conducting their own assessment of the cause of death.

Physical capability and opportunity constraints further influenced disposal practices. Farmers rarely described access to disposal infrastructure or formal guidance regarding safe disposal methods. In the absence of practical alternatives, disposal decisions were often shaped by what was feasible within available resources and local conditions. Social opportunity also influenced disposal behaviors. Religious beliefs, household practices and community traditions shaped decisions regarding the handling of dead birds and poultry waste. These social norms contributed to the continued use of disposal practices that may not align with recommended biosecurity measures. Reflective motivation was evident in farmers' efforts to minimize waste and make practical use of available resources. Disposal practices were frequently perceived as sensible and economically rational responses to poultry mortality. At the same time, automatic motivation appeared to reinforce these behaviors, with long-standing routines and habitual practices shaping how dead birds were managed.

In summary, unsafe disposal practices appeared to be driven by interacting capability, opportunity and motivation factors. While knowledge gaps regarding disease transmission were important, the behavior was also shaped by limited disposal options and socially-embedded disposal practices that had become normalized over time.

Across behaviors, the conversation events findings revealed a consistent pattern: farmers were generally able to identify the major challenges affecting poultry health and antimicrobial use, but often struggled to identify feasible solutions. The largest challenge–solution gaps were observed for chick quality and health, AMU and animal health, and knowledge and training needs (Figure 1). From a COM-B perspective, these gaps suggest that motivation to improve practices was generally present, whereas limitations in capability and, particularly physical opportunity, constrained farmers' ability to implement effective solutions. In contrast, biosecurity exhibited a relatively small challenge–solution gap (Figure 1), indicating that farmers were already experimenting with locally-adapted solutions despite resource limitations. Resilient and innovative initiatives included the use of local herbs for disease prevention and forming internal savings and lending schemes “*mukando*” to raise capital.

## Discussion

We conducted a mixed-methods baseline exploration for an intervention to strengthen biosecurity practices and prudent antimicrobial use among small-scale poultry farmers in Zimbabwe. Through a triangulation of qualitative and participatory approaches, we were able to identify key baseline behaviors as well as their determinants. We also identified potential intervention entry points.

The central finding was that indiscriminate AMU among smallholder poultry farmers in Zimbabwe did not primarily stem from ignorance or an indifference toward AMR risks. Instead, such practices served as a rational, albeit AMR-exacerbating, coping mechanism—often functioning as “behavioral insurance”— compensating for broader structural deficiencies in chick quality, preventive animal health services and disease-prevention infrastructure (21, 22). Faced with various constraints, farmers relied heavily on experiential knowledge, peer networks and traditional practices when making disease-management decisions. Although capability deficits were evident in areas such as disease diagnosis, withdrawal periods and pathogen transmission, farmers frequently demonstrated practical knowledge and adaptive problem-solving. This was particularly evident in the use of locally available alternatives for biosecurity and disease prevention. The challenge–solution gap analysis further suggested that farmers were generally able to recognize major poultry-health challenges but often lacked the resources or support required to address them effectively.

Overall, poultry-health and antimicrobial-use behaviors are responses to the interaction between structural constraints, farmer capabilities and livelihood-related motivations (13, 14, 23). A critical insight from this evaluation is that simply telling farmers “Don't use antibiotics” will not work unless identified gaps are filled. Farmers need viable alternatives: veterinary extension services, community animal-health workers and disease-recognition training. Encouraging signs include farmers' own establishment of peer networks for advice—these can be formalized into community animal health clubs or digital agri-advisory groups to disseminate proper AMU guidance.

Motivation was primarily oriented toward reducing mortality, protecting household income and maintaining flock productivity. Rather than reflecting indifference toward animal health or antimicrobial resistance, many behaviors appeared to represent attempts to manage risk under conditions of uncertainty. This was most apparent in the use of prophylactic antibiotics, which were frequently perceived as a means of protecting investments against anticipated disease losses. Similarly, decisions to sell birds before withdrawal periods had elapsed often reflected attempts to balance food-safety considerations against immediate economic needs.

The findings suggest a behavioral pathway in which constrained opportunities increase production risks and disease uncertainty, leading farmers to rely on available knowledge, informal advice networks and preventive antibiotic use to protect livelihoods. Within this system, antibiotics function not only as medicines but also as tools for managing uncertainty in environments where alternative disease-prevention options are limited (13).

Most AMR research and policy frames AMR as a knowledge problem and proposes addressing this through educational interventions (10). Our evaluation suggests that whilst these are definitely required, antibiotic use could be due to intersectional factors including, for example, poor chick quality. Conversation events suggested that farmers felt disempowered to change chick quality—it is an external issue—so they resort to coping (i.e., “medicine as insurance”). Intervening at the supply chain level could significantly reduce the perceived need for prophylactic antibiotic use, a behavioral shift with direct AMR benefits. A case study of a smallholder poultry project in Ethiopia showed that when farmers were regularly provided healthy chicks through a public-private partnership, chick survival improved and antibiotic use dropped (24). The effectiveness of this approach in the Zimbabwean context could be explored.

Intervention priorities could include sessions on waste disposal and biosecurity hygiene, with demos on setting up footbaths, proper cleaning, and low-cost adaptations like using wood ash as disinfectant, where appropriate. Of note, during both individual interviews and conversation events, farmers indicated that they were already using some of these adaptations. Therefore, instead of introducing entirely new solutions, initiatives could build on what farmers are already doing. As previously observed, farmer-led innovations can yield sustainable outcomes if properly supported (25). The intervention can, therefore, serve as both a catalyst and connector: provide the scientific input (e.g., confirm if wood ash has disinfectant properties), and let farmers drive the adaptation in their community. This aligns with participatory extension approaches which have been shown to improve adoption rates and farmer satisfaction (26). In essence, farmers should see the solutions as “ours” not “the project's”—that sense of ownership is a strong motivation for maintenance of behaviors.

Perhaps worrying is that farmers sometimes use antibiotics acting on agrovet shops' advice. This highlights the need for broader antimicrobial stewardship promotion which targets chick suppliers, agrovet shops and the farmers. Among chick suppliers and agrovet shop owners, initiatives will need to both instill and incentivise the responsible sale and promotion of antibiotics. Among farmers, initiatives will need to address the dangers of overusing antibiotics and not observing the recommended withdrawal period. The use of traditional remedies instead of antibiotics should be encouraged, but farmers need to be advised to switch to biomedical treatments when chickens are sick. This aligns with calls for stewardship approaches that are inclusive, participatory, and locally responsive (27). A systematic review of AMR interventions noted a dearth of livestock-focused behavior change interventions and emphasized that multifaceted strategies are needed (28). Our project can contribute to filling this gap by combining training, regulation (e.g., promoting AMR stewardship in agrovet shops), and supporting farmer-led monitoring of drug use. Importantly, this evaluation highlights that addressing intersectional drivers of antimicrobial use is key to designing effective One Health interventions (10).

Another prominent finding was that inequitable gender roles influenced the ability to implement biosecurity practices. Male participants acknowledged how overburdened their female counterparts were. Intervention components may need to explore the various ways in which some of the time women spend on chores can be freed up so that they adhere to biosecurity practices. Of course, this needs to be done in a manner that does not result in conflict within households. Female farmers mentioned some ways in which their male partners were supporting their business, including guarding chickens at night. These positive behaviors need to be acknowledged and encouraged. Overall, both gendered division of labor and household decision-making power shape the feasibility of adopting preventive animal health behaviors. Additionally, findings illustrate how gender intersects with time poverty and caregiving responsibilities to constrain biosecurity compliance, while also revealing opportunities for shifting norms through household-level negotiation. Further, women's agency and existing strengths (financial resourcefulness, poultry management knowledge, savings networks) are active entry points for behavior change.

A strength of our evaluation lies in the use of a mixed-methods approach including individual qualitative interviews and participatory community conversation events. Conversation events in particular, allowed quantification of issues—effectively crowdsourcing which problems are most pervasive and which solutions are favored by farmers. COM-B analysis allowed us to understand *what* behaviors were being practiced and *why*. Overall, this study contributes to the limited evidence base on livestock-sector AMR behavior change in Africa, and offers a replicable mixed-methods baseline methodology for similar One Health programmes.

A potential limitation is that translation from Shona to English, while conducted by experienced local researchers, introduces a degree of interpretive uncertainty. Additionally, conversation events gap scores are relative and context-specific: the numerical differences should be interpreted directionally rather than as precise estimates. Further, our findings may not be generalisable across all Zimbabwean villages and districts. Antibiotic use and biosecurity behaviors were likely over- or under-stated as these were based on self-reports. Finally, longitudinal data are unavailable at this baseline stage; establishing practice change will require endline evaluation.

## Conclusion

AMU among poultry farmers is closely linked to a range of behaviors, including biosecurity practices, disease diagnosis and treatment, prophylactic antibiotic use, observance of withdrawal periods and disposal of dead birds and poultry waste. Inequitable gender dynamics cut across multiple behaviors. Intervention components could include practical biosecurity training, low-cost biosecurity innovations, training on withdrawal periods, community-led disposal systems and, household workload-sharing strategies. The resultant intervention will need to be evaluated for effectiveness.

## Data Availability

The original contributions presented in the study are included in the article/supplementary material, further inquiries can be directed to the corresponding author.
