# Correction: What will it take for behavior to change? Findings from a baseline evaluation of an intervention to reduce antimicrobial use among smallholder poultry farmers in Zimbabwe

**DOI:** 10.3389/fvets.2026.1991357

**Published:** 2026-09-21

**Authors:** Patience Chenayimoyo Mushanawani, Webster Mavhu, Chenai Sipiwe Majuru, Canaan Tinashe Hodobo, Tsamwi Naume Gurira, Emily Waniwa, Kudzai Nyoka, Grace Maparo, Wasarirevu Mawere, Johannes Mubvuta, Iredale Mutengwa, Philip Zvorufura, Tariro Madzivanyika, Mackwellings Phiri, Tafadzwa Charumbira, Erica Westwood, Sarah von Humboldt-Scotland, Cèlia Ventura-Gabarró, Ahmad Wesal Zaman

**Affiliations:** 1 Department of Veterinary Technical Services, Ministry of Agriculture, Mechanisation and Water Resources Development, Harare, Zimbabwe; 2 Centre for Sexual Health and HIV/AIDS Research (CeSHHAR), Harare, Zimbabwe; 3 Department of International Public Health, Liverpool School of Tropical Medicine, Liverpool, United Kingdom; 4 Independent Consultant, Harare, Zimbabwe; 5 Malawi Liverpool Wellcome Clinical Research Programme, Blantyre, Malawi; 6 Poultry Farmers Association of Zimbabwe, Harare, Zimbabwe; 7 International Centre for Antimicrobial Resistance Solutions (ICARS), Copenhagen, Denmark

**Keywords:** antimicrobial resistance, antimicrobial use, behavior change, COM-B, one health, poultry farmers, women, Zimbabwe

The ninth author, Wasarirevu Mawere, was originally missing their surname.

The correct author list reads as follows:

Patience Chenayimoyo Mushanawani, Webster Mavhu, Chenai Sipiwe Majuru, Canaan Tinashe Hodobo, Tsamwi Naume Gurira, Emily Waniwa, Kudzai Nyoka, Grace Maparo, Wasarirevu Mawere, Johannes Mubvuta, Iredale Mutengwa, Philip Zvorufura, Tariro Madzivanyika, Mackwellings Phiri, Tafadzwa Charumbira, Erica Westwood, Sarah von Humboldt-Scotland, Cèlia Ventura-Gabarró and Ahmad Wesal Zaman

The correct **Citation** reads:

Mushanawani PC, Mavhu W, Majuru CS, Hodobo CT, Gurira TN, Waniwa E, Nyoka K, Maparo G, Mawere W, Mubvuta J, Mutengwa I, Zvorufura P, Madzivanyika T, Phiri M, Charumbira T, Westwood E, von Humboldt-Scotland S, Ventura-Gabarró C and Zaman AW (2026) What will it take for behavior to change? Findings from a baseline evaluation of an intervention to reduce antimicrobial use among smallholder poultry farmers in Zimbabwe. Front. Vet. Sci. 13:1924591. doi: 10.3389/fvets.2026.1924591

The correct **Copyright statement** reads:

© 2026 Mushanawani, Mavhu, Majuru, Hodobo, Gurira, Waniwa, Nyoka, Maparo, Mawere, Mubvuta, Mutengwa, Zvorufura, Madzivanyika, Phiri, Charumbira, Westwood, von Humboldt-Scotland, Ventura-Gabarró and Zaman. This is an open-access article distributed under the terms of the Creative Commons Attribution License (CCBY). The use, distribution or reproduction in other forums is permitted, provided the original author(s) and the copyright owner(s) are credited and that the original publication in this journal is cited, in accordance with accepted academic practice. No use, distribution or reproduction is permitted which does not comply with these terms.

The **Author Contributions** statement has been corrected to read:

PM: Conceptualization, Investigation, Methodology, Project administration, Supervision, Writing – original draft, Writing – review & editing. WMav: Formal analysis, Methodology, Validation, Writing – original draft, Writing – review & editing. CM: Conceptualization, Investigation, Methodology, Resources, Supervision, Writing – original draft, Writing – review & editing. CH: Conceptualization, Methodology, Resources, Supervision, Writing – original draft, Writing – review & editing. TG: Conceptualization, Methodology, Project administration, Resources, Writing – original draft, Writing – review & editing. EWa: Conceptualization, Methodology, Resources, Supervision, Writing – original draft, Writing – review & editing. KN: Data curation, Investigation, Methodology, Writing – original draft, Writing – review & editing. GM: Formal analysis, Investigation, Methodology, Writing – original draft, Writing – review & editing. WMaw: Conceptualization, Project administration, Resources, Writing – original draft, Writing – review & editing. JM: Formal analysis, Investigation, Methodology, Writing – original draft, Writing – review & editing. IM: Formal analysis, Investigation, Methodology, Writing – original draft, Writing – review & editing. PZ: Formal analysis, Investigation, Methodology, Writing – original draft, Writing – review & editing. TM: Formal analysis, Investigation, Methodology, Writing – original draft, Writing – review & editing. MP: Conceptualization, Formal analysis, Methodology, Writing – original draft, Writing – review & editing. TC: Conceptualization, Methodology, Validation, Writing – original draft, Writing – review & editing. EWe: Conceptualization, Funding acquisition, Methodology, Resources, Supervision, Validation, Writing – review & editing. SH-S: Conceptualization, Funding acquisition, Methodology, Resources, Supervision, Validation, Writing – original draft, Writing – review & editing. CV-G: Conceptualization, Funding acquisition, Methodology, Resources, Supervision, Validation, Writing – original draft, Writing – review & editing. AZ: Conceptualization, Funding acquisition, Methodology, Resources, Validation, Writing – original draft, Writing – review & editing.

Additionally, there was an error in the **Funding** statement as written. The funders “International Centre for Antimicrobial Resistance Solutions (ICARS)” and “International Development Research Centre (IDRC), Canada” were misspelled in the original article. The Funding statement has been updated to read:

“The author(s) declared that financial support was received for this work and/or its publication. This project (100019/100019_1100) was funded by the International Centre for Antimicrobial Resistance Solutions (ICARS). This work was also carried out with financial support from the International Development Research Centre (IDRC), Canada, and the Global AMR Innovation Fund (GAMRIF), part of UK Government's Department of Health and Social Care (DHSC). The views expressed herein do not necessarily represent those of ICARS, IDRC or its Board of Governors.”

The original version of this article has been updated.

